# Undetected Error Bounds for Hybrid Integrity Protection Using Reed–Muller Codes, Algebraic Manipulation Detection, and Universal Hashing

**DOI:** 10.3390/e28080874

**Published:** 2026-08-03

**Authors:** Buriboev Abror Shavkatovich, Akmal Abduvaitov, Jumanov Isroil, Karshiev Husan, Shavkat Buriboyev, Abbos Abduvaytov, Aziza Akhmedova, Rustam Rakhimov, Obid Mavlonov, Heung Seok Jeon

**Affiliations:** 1 Department of Artificial Intelligence, Gachon University, Seongnam 13120, Republic of Korea; abror1989@gachon.ac.kr; 2 Department of Artificial Intelligence, Samarkand State University Named After Sharof Rashidov, Samarkand 140104, Uzbekistan; abduvaitovakmal6@gmail.com (A.A.); isroil.jumanov2019@gmail.com (J.I.); h-qarshiyev@samdu.uz (K.H.); rustamjonraximov@gmail.com (R.R.); mavlonov8686@gmail.com (O.M.); 3 Department of Construction Engineering, Samarkand State Technical University, Samarkand 140143, Uzbekistan; abbosshav@gmail.com; 4 Department of IT, Samarkand Institute of Economics and Service, Samarkand 140100, Uzbekistan; abbosabduvaytov069@gmail.com; 5 Department of Exact Sciences, Kimyo International University in Tashkent, Samarkand 140143, Uzbekistan; aziza_axmedova@kiut.uz; 6 Department of Computer Engineering, AI Convergence Research Center, Konkuk University, Chungju 27478, Republic of Korea

**Keywords:** undetected error probability, algebraic manipulation detection, Reed–Muller codes, redundant coding, integrity verification, hash-based detection, coding theory

## Abstract

Ensuring information integrity requires not only reducing decoding errors but also reducing the probability that corrupted data are accepted as valid. This research presents a hybrid integrity protection system that incorporates seeded universal hash verification, algebraic manipulation detection (AMD), and a binary Reed–Muller outer code. Transmission over the binary symmetric channel BSC(p), outer encoding using RM(r, m), bounded-distance decoding, an $\varepsilon_{AMD}$-secure AMD layer, and a seeded 2-universal hash family with l-bit output define the model used in the analysis. Under explicitly stated freshness and conditional-independence assumptions, the system-level undetected error probability is upper-bounded by the residual decoder-miscorrection probability multiplied by the AMD acceptance bound and the seeded universal hash collision bound. A conservative alternative is also provided for settings in which the required conditional independence cannot be guaranteed. In this context, an explicit upper bound for the undetected error probability is derived. The outcome makes clear the different functions of outer coding and post-decoding verification and results in a direct dependency on the parameters r, m, p, and l. Finite-length Monte Carlo validation for a concrete instantiation based on RM(2, 5) complements the theoretical study and verifies that the hybrid construction offers a lower empirical undetected error probability compared to the comparable outer-only, AMD-only, and hash-only variations. The study does not propose new coding or verification primitives. Its contribution is a finite-length layered acceptance model and a Reed–Muller-specific undetected error analysis that incorporates the code weight distribution and bounded-distance decoding regions. The resulting spectrum-based bound distinguishes decoder miscorrection from the broader event of exceeding the guaranteed correction radius and is evaluated together with post-decoding verification and redundancy overhead. The model’s formal manipulation detection and collision guarantees are provided by AMD and universal hash layers, while Reed–Muller code parameters and their standard distance formulas are conventional.

## 1. Introduction

In communication systems, coding theory, and reliability-critical digital infrastructures, ensuring the integrity of transmitted and processed data continues to be a major challenge. In many real-world situations, the goal is to lower both the likelihood of decoding error and the likelihood of corrupted data being regarded as legitimate. This distinction is crucial because, in high-assurance systems, an undetected error can be even more damaging than a typical detected failure because it permits an inaccurate output to flow through the system without causing recovery or retransmission. Because of this, while designing integrity protection measures, the undetected error probability is therefore a critical performance measure. The fundamental mathematical basis for managing such failures is provided by classical error-correcting codes, but the issue of undetected acceptance cannot be entirely solved by distance-based protection alone [1].

Because of its clear algebraic structure, effective implementation, and well-understood distance qualities, linear block codes continue to be among the most crucial technologies for dependable communication. Reed–Muller codes are particularly well-known among these. Because of their explicit parameters, rich algebraic interpretation, and applicability to both theoretical and algorithmic issues, they are among the oldest and most researched code families in coding theory and continue to garner significant attention. A recent survey and related studies show research on decoding, weight distribution, threshold behavior, and performance under random errors continues to rely heavily on Reed–Muller codes [2,3,4]. Despite these advantages, the possibility of undetected acceptance cannot be eliminated by outer error-control coding alone. A decoding error may result in the acceptance of an incorrect decoded candidate even in cases where a code has strong minimum-distance properties. This encourages the introduction of extra verification methods that function after decoding and check for consistency in the decoded output. According to this more comprehensive perspective, the integrity problem naturally splits into two components: first, lowering the likelihood that the channel and decoder generate an incorrect candidate, and second, lowering the likelihood that such a candidate is recognized as legitimate. Therefore, extra post-decoding verification should be combined with distance-based protection in a mathematically adequate integrity architecture.

In this situation, two verification methods are very pertinent. The first is algebraic manipulation detection (AMD), which was developed as a primitive for identifying additive or algebraic modification of encoded data by Cramer, Dodis, Fehr, Pade, and Wichs. AMD codes have become a typical technique in integrity protection and associated cryptographic contexts because they offer formal guarantees that a modified encoding would be rejected except with a tiny probability [1,2,3,4,5]. The second is universal hashing, which was developed by Carter and Wegman and offers strict collision boundaries for seeded hash families. As a result, it offers a mathematically sound method for controlling the likelihood that a message that has been incorrectly decoded will pass a fingerprint-based consistency test [6,7]. AMD codes target algebraic manipulation, while universal hashing provides compact probabilistic verification with explicit collision guarantees.

This work examines a layered integrity protection system under an explicitly defined two-stage threat model. In the first stage, stochastic bit errors are introduced during transmission and are modeled by a binary symmetric channel, *BSC*(*p*). A binary Reed–Muller outer code and bounded-distance decoder are used to reduce the probability that these errors produce an incorrect decoded candidate. In the second stage, the decoded candidate is subjected to AMD and seeded universal hash verification. These mechanisms do not replace channel decoding; rather, they reduce the probability that a residual incorrect or algebraically modified candidate is accepted as valid. Thus, the proposed architecture distinguishes transmission reliability from acceptance integrity and combines the corresponding mechanisms within one end-to-end probabilistic model. The model is relevant to telemetry, industrial control, low-rate command transmission, safety-critical messaging, and untrusted storage or relay systems, where silent acceptance of corrupted information may be more harmful than an explicit decoding or verification failure. All coding and verification algorithms are assumed to be public. The security arguments rely on the stated AMD guarantee, the 2-universal collision property, and explicitly defined randomness assumptions rather than on secrecy of the coding architecture.

An explicit undetected error bound for the suggested hybrid approach is the paper’s primary contribution. The resultant bound under the fully stated model factors into a verification term determined by the AMD and hash layers and a residual decoding-failure term determined by the Reed–Muller code and the channel. This factorization produces a direct parameterized dependence on r, m, p, and l and makes clear the distinct responsibilities of coding and verification. Furthermore, a concrete instantiation based on RM(2, 5), a short Reed–Muller code that is also well-known in implementation-oriented decoding studies [1,2,3,4,5,6], is validated for finite length in this study.

## 2. Related Works

### 2.1. Undetected Errors After Bounded-Distance Decoding

Error-correcting codes can produce three conceptually different receiver outcomes: correct decoding, explicit decoding failure, and miscorrection to an incorrect codeword. The third outcome is especially important in integrity-sensitive systems because an incorrect codeword may be delivered without any failure indication. For a linear code used with bounded-distance decoding, a miscorrection occurs when the received vector lies inside the decoding sphere of a nontransmitted codeword. Therefore, the probability of undetected decoder error depends not only on the minimum distance but also on the number and weights of nonzero codewords and on the geometry of their decoding regions.

Classical finite-length analyses express undetected error probabilities through the weight distribution of the code. For a binary linear code, the number of received vectors of a given channel-error weight lying within a bounded-distance decoding sphere around a nonzero codeword can be counted combinatorially. Summation over the codeword weight distribution then provides a more informative characterization than a generic tail probability based only on the guaranteed correction radius. This distinction is relevant to the present work because the event that the number of channel errors exceeds the correction radius is only a necessary condition for miscorrection, not an exact characterization of it. Weight-distribution-based formulas account for the locations of competing codewords and can consequently yield substantially tighter finite-length estimates [8,9].

### 2.2. Reed–Muller Structure and Finite-Length Error Behavior

Binary Reed–Muller codes form a structured family of linear block codes defined through evaluations of low-degree Boolean polynomials. Their block length, dimension, and minimum distance are available in closed form. Beyond these basic parameters, their weight distributions, symmetries, random error behavior, and list-decoding properties have been studied extensively. In particular, existing results show that the weight spectrum and distribution of low-weight RM codewords contain information not captured by minimum distance alone.

These properties are important for undetected error analysis. Minimum distance determines the guaranteed correction radius, but the probability of being miscorrected to another codeword depends on how many nonzero codewords occur at each weight and on the channel probability of reaching their decoding neighborhoods. The present study therefore uses the Reed–Muller weight distribution to refine the generic binomial-tail bound. For the concrete $RM\left(2,5\right)$ example, the relevant weight multiplicities can be determined exactly, permitting a code-specific finite-length evaluation. For longer or higher-order RM codes for which a complete weight enumerator is unavailable or expensive to compute, partial spectra, enumerated spectra, or established upper bounds can be used [10,11,12].

### 2.3. AMD and Universal Hash Verification

AMD codes address algebraically structured manipulation by encoding a source with randomness such that a nonzero admissible offset produces another valid encoding only with bounded probability. Universal hashing addresses a different verification problem: for distinct messages, a randomly selected hash function from a universal family produces the same output only with a bounded collision probability. AMD has also been extended to broader classes of algebraic manipulation functions, demonstrating that its security guarantee is defined relative to a specified tampering family rather than arbitrary unrestricted corruption [13,14,15]. Universal and almost-universal hash families have similarly been studied as components of authentication and integrity mechanisms with explicit collision bounds (see Table 1).

In the present framework, neither mechanism is treated as an error-correcting code. They are post-decoding acceptance mechanisms. The AMD layer tests algebraic consistency of the protected representation, while the universal hash layer provides an independently parameterized consistency check for a recovered message. Their contribution is therefore evaluated conditionally on the decoder producing an incorrect candidate.

### 2.4. Joint Coding and Verification Architectures

Combined correction-and-detection architectures have previously been studied using error-correcting codes with unused syndromes, CRC checks, parity checks, hashes, MACs, or other verification mechanisms. In such designs, the outer code determines the probability of decoder failure or miscorrection, while an additional mechanism determines whether an incorrect output is detected. Finite-length analyses of combined error correction and detection show that decoding-sphere geometry and codeword weight distributions can be used to calculate undetected error probability more precisely than a correction-radius tail alone [16,17,18]. Other studies of coding under natural and malicious faults have analyzed error masking or integrity verification, but often under threat models, code constructions, or authentication assumptions different from those considered here.

The present work is not distinguished merely by placing three known blocks in sequence. Its intended distinction is the formulation of one finite-length acceptance model that simultaneously identifies:

1. Outer-code miscorrection under a specified channel and decoder;
2. AMD acceptance of an admissibly modified or incorrect representation;
3. Universal hash acceptance of a distinct message;
4. Total verification and coding redundancy.

Unlike a generic product of a binomial tail and verification parameters, the refined analysis introduced below replaces the channel-tail term with an RM-specific decoding region expression based on the code’s weight spectrum. The manuscript also compares protection modes under matched payload and matched redundancy conditions, rather than comparing only their raw undetected error probabilities.

### 2.5. Research Gap and Position of the Present Study

Existing studies provide strong results for the individual components considered here. Reed–Muller research characterizes code structure, decoding behavior, and weight distributions. AMD research formalizes detection guarantees for algebraic manipulation, while universal hashing provides bounded collision probabilities. Classical coding literature also provides finite-length analyses of undetected decoder errors and combined error-correction/error-detection systems. Accordingly, the novelty of the present study does not lie in introducing any of these individual mechanisms. The specific gap addressed here is narrower. We consider a fully specified finite-length system in which an outer Reed–Muller decoder may produce an incorrect tagged candidate and in which that candidate is subsequently evaluated by two separately parameterized verification mechanisms. The study links the geometry of Reed–Muller decoding regions to the probability of final acceptance, decomposes total redundancy into outer-code and verification components, and evaluates the resulting trade-off under common payload and redundancy constraints. Compared with a generic correction-radius analysis, the principal theoretical addition is an RM-specific bound based on the weight distribution of the selected Reed–Muller code [19,20]. This refinement distinguishes the probability that the BSC produces more than t errors from the smaller and operationally relevant probability that the received vector falls into the decoding region of an incorrect RM codeword. The resulting miscorrection term is then combined with the explicitly stated post-decoding verification guarantee. Thus, the paper’s contribution is a code-specific finite-length acceptance analysis rather than a claim of a new code or verification primitive.

The contribution of this study is not a new Reed–Muller code, AMD construction, universal hash family, or decoding algorithm. Instead, the work provides a finite-length system analysis of layered integrity acceptance. The contributions are threefold. First, we formulate a unified integrity experiment that separates stochastic channel corruption, bounded-distance decoder failure or miscorrection, and post-decoding verification. The model explicitly defines the channel, decoder, adversary capabilities, verification randomness, and conjunctive acceptance rule. Second, we derive two levels of undetected error analysis. A general bound separates the residual decoder-miscorrection probability from the conditional verification-acceptance probability. We then specialize the decoder term to binary Reed–Muller codes by using their finite-length weight distribution and bounded-distance decoding regions. This produces an RM-specific bound that is tighter than the generic binomial-tail expression based solely on the minimum distance. Third, we evaluate the integrity–redundancy trade-off across outer-only, AMD-assisted, hash-assisted, and hybrid configurations. The revised evaluation varies the Reed–Muller parameters, AMD security level, and hash length and includes matched-payload and matched redundancy comparisons together with computational and storage overhead.

### 2.6. Unified Threat Model and Intended Application Scenario

The proposed framework addresses an end-to-end integrity problem involving two logically distinct sources of corruption. The first is stochastic channel corruption, represented in this study by a binary symmetric channel, BSC(p). During transmission, each bit of the Reed–Muller codeword is independently inverted with probability p. This component models non-adversarial disturbances such as noise, transient link faults, unreliable storage elements, or random bit errors occurring in a communication or relay channel. The role of the outer Reed–Muller code is to correct error patterns within its guaranteed decoding radius and thereby reduce the probability that an incorrect tagged payload reaches the verification stage. The second source of risk is an integrity-acceptance threat. Even when the number of channel errors exceeds the guaranteed correction capability of the outer code, the decoder may return an incorrect candidate rather than the originally transmitted tagged message. In addition, in applications that include an untrusted relay, storage node, interface, or processing stage, the tagged representation may be subjected to a deliberate algebraic modification. The AMD layer is introduced to detect nonzero additive manipulations of the protected representation, whereas the seeded universal hash layer provides an additional probabilistic consistency check for distinguishing the original message from an incorrect candidate. These two threats are therefore neither identical nor treated as competing explanations for the same event. They act at different logical stages of the system. Random channel errors act on the transmitted Reed–Muller codeword before decoding. AMD and hash verification act after decoding by determining whether the recovered candidate should be accepted as valid. The system-level undetected error event occurs only when the receiver obtains an incorrect source-message candidate and the applicable post-decoding verification conditions nevertheless accept that candidate.

The baseline analytical setting assumes that the transmitted Reed–Muller codeword is corrupted by the BSC(p). Under this setting, the AMD and hash layers provide post-decoding verification of residual incorrect candidates. The extended threat interpretation additionally permits an algebraic modification of the AMD-protected representation at an untrusted processing, storage, or relay stage. The present theorem is derived for the explicitly stated stochastic-channel experiment, while the AMD security definition provides the verification guarantee against admissible nonzero algebraic offsets. The manuscript does not claim that a memoryless BSC fully represents an adaptive adversarial channel.

The system parameters and algorithms are assumed to be public. In particular, an adversary may know the Reed–Muller code RM(r,m), the bounded-distance decoding rule, the AMD construction, and the selected universal hash family. Security is not based on keeping these algorithms secret. The AMD randomness and the hash seed are generated independently according to the distributions specified by the corresponding constructions. In the present implementation, the hash seed is carried as part of the tagged representation so that the receiver can reproduce the hash computation. Consequently, the seed is not treated as a long-term secret key. Its purpose is to select a member of the 2-universal family independently of the protected message and admissible corruption event. Any stronger threat model involving adaptive seed-dependent manipulation, seed reuse, or compromise of the random-number generator would require a separate analysis.

The framework is intended for systems in which silently accepting corrupted information is more critical than merely detecting a decoding failure. Representative scenarios include low-rate telemetry links, industrial command and control messages, safety-critical status transmission, remote sensing data, and storage or relay systems in which a decoded output is passed to a subsequent process only after integrity verification. For example, a telemetry packet may be affected by random link noise during transmission and may subsequently pass through a relay or storage component that is not fully trusted. In such a setting, the outer Reed–Muller code improves transmission reliability, while the AMD and universal hash layers reduce the probability that an incorrect recovered message is silently treated as valid (see Table 2).

The present work consequently adopts a layered rather than a single-threat interpretation. Reed–Muller coding addresses the probability of producing an incorrect candidate under stochastic channel corruption. AMD verification addresses admissible algebraic manipulation, and universal hashing limits the probability that a distinct candidate satisfies the hash consistency condition [21,22]. Their combination is motivated by the fact that error correction and integrity acceptance are different system functions: successful decoding attempts to recover the transmitted tagged object, whereas post-decoding verification determines whether the resulting object can be trusted.

## 3. Mathematical Model and Notation

This section introduces the concrete mathematical model used throughout the paper. In contrast to generic layered integrity architectures, the present work focuses on a fully specified setting consisting of a binary symmetric channel, a binary Reed–Muller outer code, bounded-distance decoding, an explicit algebraic manipulation detection layer, and a seeded universal hash verification layer. This choice allows the undetected error probability to be expressed directly in terms of the code parameters and the channel crossover probability (Table 3). Reed–Muller codes are particularly suitable for this purpose because their length, dimension, and minimum distance are available in closed form.

### 3.1. Channel Model and Notation

Let $F_{2}$ denote the binary field, and let the source message be a vector

(1) $$M\in F_{2}^{k}.$$

The BSC(p) represents stochastic, non-adversarial corruption of the transmitted Reed–Muller codeword. It is not intended to represent a fully adaptive manipulation adversary. The AMD threat model is defined separately at the tagged-representation level. Consequently, the random error vector E and an admissible algebraic manipulation offset Δ are different mathematical objects: E acts on the transmitted codeword through the communication channel, whereas Δ represents a nonzero algebraic modification considered under the security definition of the AMD construction. The baseline theorem evaluates residual incorrect candidates caused by E; the AMD and hash layers then bound the probability that such a candidate passes post-decoding verification. The transmitted codeword is sent through a binary symmetric channel BSC(p), where each coordinate is flipped independently with probability p, 0<p<1/2. Thus, if

(2) $$X\in F_{2}^{n}$$

is the transmitted codeword and

(3) $$E=(E_{1},\ldots ,E_{n})\in F_{2}^{n}$$

is the random error vector with i.i.d. components $E_{i}∼Bernoulli(p)$, then the received word is

(4) Y=X⊕E.

The Hamming weight of a vector $x\in F_{2}^{n}$ is denoted by $w_{H}(x)$, and the Hamming distance between $x,z\in F_{2}^{n}$ is denoted by $d_{H}(x,z)$. Since the channel is binary and memoryless, the distribution of $w_{H}(E)$ is binomial:

(5) Pr{wH(E)=i}=nipi(1−p)n−i,  i=0,1,…,n.

This binomial tail will play a central role in the undetected error bounds developed later.

#### Probability Space and Order of Random-Variable Generation

The system analysis is defined over a joint probability space containing the source message, AMD randomness, universal hash seed, channel-error vector, and decoder output. The order in which these random variables are generated is important because the post-decoding verification bound depends on their conditional relationships.

The experiment proceeds as follows:

1. A source message M is selected according to a specified source distribution.
2. Fresh AMD randomness R is sampled according to the AMD construction.
3. The AMD-protected representation is generated from M  and R.
4. A hash seed S is sampled independently of M and R from the prescribed seed space.
5. The hash tag $T_{H}=h_{S}\left(M\right)$ is computed.
6. The complete tagged representation is embedded into the information coordinates of the Reed–Muller encoder.
7. A channel-error vector E is generated according to the memoryless BSC(p), independently of R and S.
8. The receiver applies bounded-distance decoding and obtains either a decoder failure or a candidate tagged representation $\hat{T}$.
9. If a candidate is produced, the receiver applies the AMD and universal hash verification rules.

Let D denote the event that the decoder outputs an incorrect but syntactically valid tagged candidate. Let $A_{AMD}$ denote the event that this candidate passes the AMD verification rule, and let $A_{H}$ denote the event that it passes the universal hash verification rule. The system-level undetected error event is

(6) $$U=D\cap A_{AMD}\cap A_{H}.$$

The baseline analysis assumes that R, S, and E are generated independently according to their prescribed distributions. It further assumes that the incorrect candidate to which the verification guarantees are applied is fixed before the fresh hash seed is evaluated, or equivalently, that conditioning on the decoder event and the AMD acceptance event does not bias the distribution of S. These assumptions are stated explicitly because the product-form verification bound is not valid under arbitrary dependence.

### 3.2. Reed–Muller Outer Coding

As the outer error-control code, we use the binary Reed–Muller code RM(r,m), where 0≤r≤m. This code has length

$$n=2^{m},$$

dimension

kRM=∑i=0rmi,

and minimum distance

$$d=2^{m-r}.$$

These parameters are classical and provide the main reason for selecting Reed–Muller codes in the present work: they allow the correction radius and residual decoding-failure region to be described explicitly. In addition, Reed–Muller codes admit efficient decoding algorithms and remain one of the most studied structured code families in modern coding theory.

The receiver is assumed to use a bounded-distance decoder for RM(r,m). Its guaranteed correction radius is

(7) $$t=\left\lfloor \frac{d-1}{2}\right\rfloor =\left\lfloor \frac{2^{m-r}-1}{2}\right\rfloor =2^{m-r-1}-1.$$

Accordingly, every received vector Y satisfying

$$w_{H}(E)\leq t$$

is decoded correctly. Any decoding failure or incorrect decoding can therefore occur only when

$$w_{H}(E)>t.$$

This observation provides the first structural ingredient in system-level reliability analysis.

### 3.3. AMD Verification Layer

To detect structured additive manipulations that may survive outer decoding, we incorporate an algebraic manipulation detection (AMD) layer. AMD codes were introduced by Cramer, Dodis, Fehr, Padro, and Wichs as keyless mechanisms for detecting additive tampering, with security defined through the probability that a nonzero additive offset transforms a valid encoding into another valid encoding.

Formally, let $C_{AMD}=AMDEnc\left(M;R\right)$ denote the AMD-protected representation generated using fresh randomness R. An admissible algebraic adversary selects a nonzero additive offset Δ from the alphabet of the AMD construction and causes the verifier to receive $C_{AMD}+\Delta$. The adversary may know the AMD construction and all public system parameters but does not control the fresh encoder randomness R before selecting an offset in the security experiment assumed here. The AMD construction is $\varepsilon_{AMD}$-secure if, for every admissible nonzero Δ,

(8) $$\underset{R}{Pr}\;\left[\mathrm{AMDDec}\left(AMDEnc\left(M;R\right)+\Delta \right)\neq ⊥\right]\leq \varepsilon_{AMD}$$

Here, ⊥ denotes rejection. This guarantee applies to algebraically structured manipulation of the AMD-protected representation and should not be interpreted as a complete model of an arbitrary adaptive communication-channel adversary.

### 3.4. Seeded Universal Hash Verification

As a second verification layer, we use a seeded universal hash family in the sense of Carter and Wegman. Let

(9) $$H=\{h_{s}:M\to \{0,1{\}}^{l}{\}}_{s\in S}$$

be a 2-universal family of hash functions with l-bit output. The hash family and its evaluation algorithm are public. A seed S is sampled independently of M and the AMD randomness R, and the corresponding hash value is $T_{h}=h_{S}\left(M\right)$. In the present construction, S is included in the tagged representation to allow receiver-side verification; therefore, it is not treated as a secret authentication key. The collision guarantee applies when S is sampled according to the prescribed seed distribution and is not maliciously selected as a function of a competing message pair. Adaptive manipulation after observing or controlling the seed, repeated seed reuse, and compromised seed generation are outside the present theorem and are identified as limitations. This means that for any two distinct messages $M\neq M^{\prime }$,

$$\underset{S}{Pr}\{h_{S}(M)=h_{S}(M^{\prime })\}\leq 2^{-l},$$

when the seed s is chosen uniformly at random. Universal hashing is especially appropriate here because it gives a direct and rigorous upper bound on the probability that an incorrect decoded message collides with the stored integrity tag.

For a given source message M, the hash tag is

$$\tau_{H}=h_{S}(M),$$

and the hash-augmented representation is

$$T_{H}(M;s)=(M,s,\tau_{H}).$$

At the receiver, a candidate message $\hat{M}$ passes the hash test only if

$${\hat{\tau }}_{H}=h_{s}(\hat{M}).$$

The seed S is sampled independently of the source message, AMD randomness, channel-error vector, and admissible manipulation event. For any fixed pair of distinct messages $M\neq \hat{M}$, the 2-universal property gives

(10) $$\underset{S}{Pr}\;\left[h_{S}\left(M\right)=h_{S}\left(\hat{M}\right)\right]\leq 2^{-l}.$$

The sequential theorem below additionally requires that conditioning on the decoder event D and on the AMD acceptance event $A_{AMD}$ does not alter the prescribed distribution of S. Under this condition,

(11) $$Pr\;(A_{H^{\prime }}|DA_{AMD})\leq 2^{-l}.$$

This condition is satisfied when the hash seed is fresh, sampled independently, and not chosen adaptively as a function of the incorrect candidate or AMD verification outcome. It may fail under malicious seed selection, correlated seed generation, adaptive seed-dependent manipulation, or repeated seed reuse combined with information leakage.

### 3.5. Combined Tagged Representation and Transmission

The proposed hybrid construction combines both verification mechanisms before outer encoding. Let the tagged message be

(12) $$T(M;R,s)=(M,\;R,\;f(M,R),\;s,\;h_{s}(M)).$$

Assume that this tagged object is embedded into the information part of the Reed–Muller encoder, yielding the transmitted codeword

(13) $$X={Enc}_{RM}(T(M;R,s))\in F_{2}^{n}.$$

After transmission through BSC(p), the receiver obtains

Y=X⊕E

and applies bounded-distance decoding to produce a candidate tagged vector

(14) $$\hat{T}=(\hat{M},\hat{R},{\hat{\tau }}_{AMD},\hat{s},{\hat{\tau }}_{H}).$$

The receiver accepts the decoded output only if both verification conditions hold:

(15) $${\hat{\tau }}_{AMD}=f\left(\hat{M},\hat{R}\right)\;\;\mathrm{and}\;{\hat{\tau }}_{H}=h_{\hat{s}}\left(\hat{M}\right).$$

In the implementation considered here, the seed κ is transmitted as part of the tagged object, so the hash check remains fully specified and reproducible to the receiver. The resulting undetected error event is therefore the event that an incorrect decoded candidate is produced and simultaneously passes both the AMD and hash verification stages.

### 3.6. Performance Metric

The main performance metric is the probability of undetected error,

(16) $$P_{ue}=Pr\{\hat{M}\neq M\;\mathrm{and}\;\mathrm{the}\;\mathrm{receiver}\;\mathrm{accepts}\}.$$

This is a stricter event than decoder failure alone. A decoder error does not yet imply system failure, because the incorrect candidate may still be rejected by the AMD or hash verification stages. To avoid ambiguity, four receiver outcomes are distinguished throughout the manuscript. A decoder failure occurs when the bounded-distance decoder does not return a candidate codeword and outputs ⊥. A decoder miscorrection occurs when the decoder returns a codeword or tagged representation different from the transmitted one. A detected error occurs when channel corruption or decoder miscorrection is identified either through decoder failure or through failure of at least one post-decoding verification check. A rejected candidate is any decoder output that is not accepted because the AMD check, the hash check, or a syntactic validity check fails. Finally, an undetected error occurs only when an incorrect source-message candidate is delivered as valid after all enabled verification checks have accepted it. Accordingly, the purpose of the hybrid architecture is not only to reduce decoding error through outer coding, but also to reduce the probability that a residual decoding error is accepted as valid. The analysis in Section 5 shows that these two effects can be separated explicitly in the fully specified model introduced above.

## 4. Proposed Hybrid Integrity Protection Scheme

This section specifies the hybrid integrity protection scheme under the mathematical model and notation introduced in Section 3.

**Figure 1:**
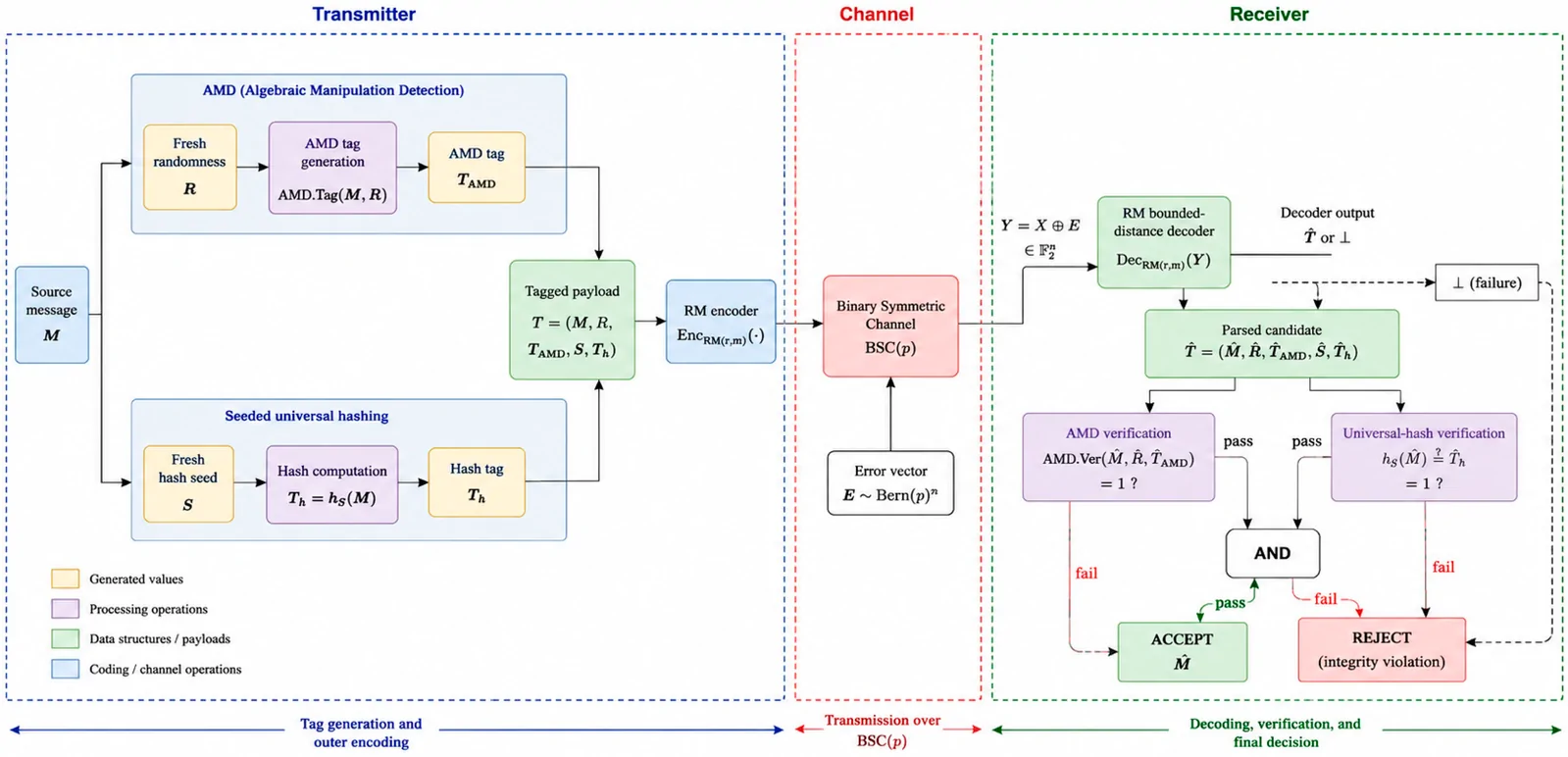
End-to-end structure of the proposed layered integrity protection system.

The construction combines three components: a binary Reed–Muller outer code, an AMD tagging layer, and a seeded universal hash verification layer as shown in Figure 1. The purpose of the architecture is to ensure that a decoding error at the outer-code level does not automatically imply acceptance of an incorrect source message. Instead, a decoded candidate is accepted only if it also satisfies the AMD and hash consistency conditions.

### 4.1. Encoder Structure

Let $\;M\in F_{2}^{k}$ be the source message. Before outer encoding, the message is augmented by two verification layers. First, an AMD randomness variable R is generated, and the AMD tag is computed as $\tau_{AMD}=f(M,R),$ where f is the AMD encoding function. Second, a hash seed s is selected and the universal hash tag is computed as $\tau_{H}=h_{s}(M),$ where $h_{s}$ is a member of the seeded 2-universal hash family introduced in Section 2.

The resulting tagged object is

(17) $$T(M;R,s)=(M,\;R,\;f(M,R),\;s,\;h_{s}(M)).$$

Let L denote the binary length of this tagged representation. The tagged vector is then embedded into the information part of the outer Reed–Muller encoder. Denoting the binary Reed–Muller encoding map by ${Enc}_{RM}:F_{2}^{L}\to F_{2}^{n},$ the transmitted codeword is

(18) $$X={Enc}_{RM}(T(M;R,s)),$$

where $n=2^{m}$ for the chosen code RM(r,m).

Thus, the encoder consists of two logically distinct stages:

1. Message augmentation, which adds integrity-check information.
2. Outer coding, which provides distance-based protection against random channel perturbations.

This separation is central to the hybrid design, because the verification layers and the outer code serve different mathematical purposes.

Encoder-side sequence:

1. Generate source message M.
2. Generate fresh AMD randomness R.
3. Compute the AMD-protected representation.
4. Generate a fresh hash seed S, independently of M and R.
5. Compute $T_{h}=h_{S}\left(M\right)$.
6. Form the tagged representation.
7. Embed the tagged representation into the information positions of RM(r,m).
8. Transmit the resulting codeword through BSC(p).

### 4.2. Transmission Model

The codeword X is transmitted through the BSC(p) defined in Section 3.1, and the receiver observes Y according to Equation (4).

The outer Reed–Muller code is intended to correct sufficiently small perturbations. However, if the noise pattern lies outside the guaranteed correction radius of the bounded-distance decoder, then the receiver may output an incorrect tagged candidate. The role of the verification layers is precisely to reduce the probability that such an incorrect candidate is nevertheless accepted.

### 4.3. Decoder and Verification Procedure

At the receiver, the first stage is bounded-distance decoding with respect to the outer Reed–Muller code RM(r,m). Let

(19) $${Dec}_{RM}(Y)$$

denote the decoder output. If the received vector lies within Hamming distance

(20) $$t=2^{m-r-1}-1$$

of the transmitted codeword, then the decoder returns the correct tagged vector. Otherwise, the decoder may fail or return an incorrect candidate. Suppose the decoder outputs

(21) $$\hat{T}=(\hat{M},\hat{R},{\hat{\tau }}_{AMD},\hat{s},{\hat{\tau }}_{H}).$$

The receiver accepts this candidate only if both of the following conditions hold:

$${\hat{\tau }}_{AMD}=f(\hat{M},\hat{R}),$$

and

$${\hat{\tau }}_{H}=h_{\hat{s}}(\hat{M}).$$

Therefore, the acceptance rule is conjunctive:

$$\mathrm{Accept}\;\hat{M}\;⟺\;({\hat{\tau }}_{AMD}=f(\hat{M},\hat{R}))\;\mathrm{and}\;({\hat{\tau }}_{H}=h_{\hat{s}}(\hat{M})).$$

If either verification condition fails, the decoded output is rejected. In that case, the system may declare an integrity violation, request retransmission, or invoke a higher-layer recovery procedure, depending on the application context. These operational responses are external to the coding model itself and are therefore not included in the mathematical analysis.

Receiver-side sequence:

1. Apply bounded-distance decoding.
2. Reject immediately if the decoder declares failure or if the output cannot be parsed as a valid tagged representation.
3. Extract the candidate message, AMD data, seed, and hash tag.
4. Apply the AMD verification condition.
5. Recompute the universal hash using the recovered seed and candidate message.
6. Accept only when both checks succeed.
7. Otherwise, return ⊥ or declare an integrity violation.

### 4.4. Protection Modes Considered in the Paper

For analytical comparison, the proposed framework includes four protection modes derived from the same general architecture.

Outer-code-only mode. In the baseline mode, only the source message is encoded by the outer Reed–Muller code. No AMD or hash verification is applied after decoding. Thus, any incorrectly decoded source message is accepted automatically.

AMD-only mode. In this variant, the encoded payload is

(22) $$T^{\left(A\right)}=(M,R,T_{AMD}).$$

After outer decoding, the receiver accepts the candidate only if the AMD consistency condition is satisfied.

Hash-only mode. In this variant, the encoded payload is

(23) $$T^{\left(H\right)}=(M,S,T_{h}).$$

After outer decoding, the receiver accepts the candidate only if the hash consistency condition holds.

Hybrid mode. In the full hybrid construction, both verification layers are included:

(24) $$T^{\left(AH\right)}=(M,R,T_{AMD},S,T_{h}).$$

A decoded candidate is accepted only when it passes both checks.

These four modes make it possible to compare the separate and joint effects of AMD and universal hash verification under the same outer-code and channel model.

Under the construction above, the system-level undetected error event is defined as

(25) $$E_{ue}=\{\hat{M}\neq M\;\mathrm{and}\;\mathrm{the}\;\mathrm{receiver}\;\mathrm{accepts}\;\hat{M}\}.$$

This event is stronger than decoder error alone. A decoding error produces a system-level failure only if the incorrect candidate also survives the verification stage associated with the chosen protection mode. Consequently, the hybrid architecture separates two logically distinct failure mechanisms:

- The failure of the outer code to recover the correct tagged object.
- The failure of the verification stage to reject an incorrect decoded candidate.

This separation is exactly what enables the factorized undetected error to bound, derived in Section 5.

### 4.5. Redundancy Structure of the Construction

The hybrid scheme introduces redundancy at two different levels. If L is the binary length of the tagged object T(M;R,κ), then the verification redundancy is

(26) $$\rho_{ver}=L-k,$$

while the outer-code redundancy is

(27) $$\rho_{out}=n-L.$$

Hence, the total redundancy is

(28) $$\rho_{tot}=n-k=\rho_{ver}+\rho_{out}.$$

This decomposition is important because the two redundancy terms play different roles. The outer-code redundancy determines the distance-based correction capability of the Reed–Muller layer, whereas the verification redundancy determines the strength of the AMD and hash checks. The proposed architecture therefore allows reliability improvement to be distributed between coding protection and verification protection in a mathematically transparent way.

## 5. Theoretical Analysis

This section derives upper bounds on the probability of undetected error defined in Equation (27). Section 5.1 establishes the sequential verification bound under explicit randomness and conditional-independence assumptions. Section 5.2 derives a general system-level bound using the bounded-distance correction radius in Equation (10) and the BSC tail in Equation (5). Section 5.3 then refines the decoder term using Reed–Muller-specific weight distribution and decoding region information.

### 5.1. Explicit Bound for the Hybrid Construction

**Lemma 1.**  *Let* D *be the event that the decoder outputs an incorrect tagged candidate. Let* $A_{AMD}$ *and* $A_{H}$ *denote acceptance by the* AMD *and universal hash checks, respectively. Assume that:*

1. *For every admissible incorrect decoded candidate,*
  (29) $$Pr\left(A_{AMD}|D\right)\leq \varepsilon_{AMD};$$
  *the hash seed is sampled independently of the variables determining* D *and* $A_{AMD}$;
2. *Conditioned on* D *and* $A_{AMD}$*, the candidate message pair remains fixed and distinct before evaluation over the hash seed;*
3. *The hash family is seeded 2-universal with*  l*-bit output. Then,*
  (30) $$Pr\left(A_{AMD}\cap A_{H}|D\right)\leq \varepsilon_{AMD}2^{-l}.$$

**Proof.**  By the conditional chain rule,

(31) $$Pr\left(A_{AMD}\cap A_{H}|D\right)=Pr\left(A_{AMD}|D\right)Pr\;(A_{H^{\prime }}|DA_{AMD}).$$

The AMD security assumption gives

(32) $$Pr\left(A_{AMD}|D\right)\leq \varepsilon_{AMD}.$$

By the independence and freshness assumptions on the hash seed, conditioning on D and $A_{AMD}$ does not alter the prescribed distribution of S. The incorrect decoded message remains distinct from the transmitted message, so the 2-universal collision property gives

(33) $$Pr\;(A_{H^{\prime }}|DA_{AMD})\leq 2^{-l}.$$

Substituting Equations (32) and (33) into Equation (31) yields

(34) $$Pr\left(A_{AMD}\cap A_{H}|D\right)\leq \varepsilon_{AMD}2^{-l}.$$

This proves the claim. □

**Theorem 1.**  *General hybrid undetected error bound under sequential verification.*

Let C=RM(r, m) be a binary Reed–Muller code of length with minimum distance and bounded-distance decoding radius. Assume transmission over $BSC\left(p\right)(p)$. Let D denote the event that the bounded-distance decoder outputs an incorrect tagged candidate. Suppose the AMD and universal hash verification layers satisfy the assumptions of Lemma 1. Then,

(35) $$P_{UE}=Pr\left(D\cap A_{AMD}\cap A_{H}\right)\leq Pr\left(D\right)\varepsilon_{AMD}2^{-l}.$$

Since a bounded-distance decoder is guaranteed to return the transmitted codeword whenever $wt\left(E\right)\leq t$,

(36) $$Pr\left(D\right)\leq Pr\left\{wt\left(E\right)>t\right\}.$$

Therefore,

(37) PUE≤εAMD2−l∑j=t+1nnjpj1−pn−j.

**Proof.**  By definition, the system-level undetected error event is

$$U=D\cap A_{AMD}\cap A_{H}.$$

Applying conditional probability,

(38) $$Pr\left(U\right)=Pr\left(D\right)Pr\left(A_{AMD}\cap A_{H}|D\right).$$

Lemma 1 gives

(39) $$Pr\left(A_{AMD}\cap A_{H}|D\right)\leq \varepsilon_{AMD}2^{-l}.$$

Consequently,

(40) $$Pr\left(U\right)\leq Pr\left(D\right)\varepsilon_{AMD}2^{-l}.$$

For bounded-distance decoding, the decoder is guaranteed to recover the transmitted codeword whenever the channel-error weight does not exceed t. Therefore,

$$D\subseteq \left\{wt\left(E\right)>t\right\},$$

and hence,

(41) $$Pr\left(D\right)\leq Pr\left\{wt\left(E\right)>t\right\}.$$

Since E is generated by BSC(p), its Hamming weight is binomially distributed:

(42) PrwtE>t=∑j=t+1nnjpj1−pn−j.

Combining Equations (40)–(42) yields Equation (37). □

**Theorem 2.**  *RM-spectrum-based hybrid undetected error bound. Let* C=RM(r,m) *be used over* BSC(p) *with a bounded-distance decoder of radius* t*. Let* $A_{w}$ *be the weight distribution of* C*, and let* $P_{misc}^{RM}\left(p\right)$ *be defined by Equation (43). Suppose that, conditional on a fixed incorrect decoded tagged candidate, the joint probability that the candidate passes both verification layers is at most* $\varepsilon_{ver}$*. Then, *

(43) $$P_{UE}^{hyb}\leq P_{misc}^{RM}\left(p\right)\varepsilon_{ver}.$$

*Under the additional assumptions required to establish independent AMD and hash verification bounds,*

$$\varepsilon_{ver}\leq \varepsilon_{AMD}2^{-l},$$

*and therefore,*

(44) $$P_{UE}^{hyb}\leq \varepsilon_{AMD}2^{-l}\sum_{w=d_{min}}^{n}A_{w}\sum_{j=max\left(0,w-t\right)}^{min\left(n,w+t\right)}N_{t}\left(n,w,j\right)p^{j}{\left(1-p\right)}^{n-j}.$$

**Proof.**  Because C is linear and the BSC is symmetric, the all-zero codeword may be assumed to have been transmitted. A bounded-distance miscorrection occurs when the received vector lies within radius t of a nonzero codeword c∈C. For a codeword of weight w, $N_{t}(n,w,j)$ counts the weight-j vectors lying within its decoding sphere. Each such vector occurs with BSC probability $p^{j}{\left(1-p\right)}^{n-j}$. Summing over channel weights, codeword weights, and the corresponding multiplicities $A_{w}$ yields $P_{misc}^{RM}\left(p\right)$.

An undetected system error requires both a bounded-distance miscorrection and acceptance by the enabled verification mechanisms. By conditioning on the incorrect decoded candidate and applying the uniform joint verification bound $\varepsilon_{ver}$,

(45) $$P_{UE}^{hyb}=\sum_{\hat{T}\neq T}P\left(\hat{T}\;\mathrm{is}\;\mathrm{decoded}\right)P\left(\hat{T}\;\mathrm{is}\;\mathrm{accepted}|\hat{T}\;\mathrm{is}\;\mathrm{decoded}\right)\leq \varepsilon_{ver}\sum_{\hat{T}\neq T}P\left(\hat{T}\;\mathrm{is}\;\mathrm{decoded}\right)=P_{misc}^{RM}\left(p\right)\varepsilon_{ver}.$$

The final expression follows when the revised verification theorem establishes

(46) $$\varepsilon_{ver}\leq \varepsilon_{AMD}2^{-l}.$$

□

### 5.2. Refined Finite-Length Bound Based on Decoder Miscorrection

The correction-radius tail used in the general theorem is convenient because it depends only on the code length, minimum distance, and channel crossover probability. However, it is generally not equal to the decoder-miscorrection probability. For a bounded-distance decoder, the event

$$\left\{W>t\right\}$$

includes both incorrect decoding and explicit decoder failure. Consequently,

(47) $$P_{miscorr}\leq Pr\left\{W>t\right\}.$$

A tighter system-level representation is obtained by conditioning directly on the decoder-miscorrection event $D_{mis}$:

(48) $$P_{UE}=Pr\left(D_{mis}\right)Pr\left(A_{AMD}\cap A_{h}|D_{mis}\right).$$

Let

(49) $$P_{miscorr}=Pr\left(D_{mis}\right)$$

and

(50) $$P_{ver,acc|miscorr}=Pr\left(A_{AMD}\cap A_{h}|D_{mis}\right).$$

Then,

(51) $$P_{UE}=P_{miscorr}P_{ver,acc|miscorr}.$$

Under the sequential verification assumptions of Lemma 1,

(52) $$P_{ver,acc|miscorr}\leq \varepsilon_{AMD}2^{-l},$$

and therefore

(53) $$P_{UE}\leq P_{miscorr}\varepsilon_{AMD}2^{-l}.$$

Equation (53) is tighter than the generic correction-radius result whenever $P_{miscorr}$ is evaluated or upper-bounded more accurately than $Pr\left\{W>t\right\}$.

Reed–Muller-specific miscorrection bound. Let C=RM(r,m), and let $A_{w}$ denote the number of codewords of Hamming weight w.

Assume that the all-zero codeword is transmitted, which is valid for a linear code over a symmetric channel. For a nonzero codeword c of weight w, define the bounded-distance decoding sphere

(54) $$B_{t}\left(c\right)=\left\{y\in F_{2}^{n}:d_{H}\left(y,c\right)\leq t\right\}.$$

A miscorrection to c occurs only when

$$Y\in B_{t}\left(c\right).$$

Therefore,

(55) $$P_{miscorr}=\sum_{c\in C\setminus \left\{0\right\}}Pr\left\{Y\in B_{t}\left(c\right)\right\},$$

provided the bounded-distance decoding spheres are disjoint and the decoder outputs a codeword only when the received vector lies in one such sphere. If the implementation uses another decoder rule, use ≤ rather than equality.

For a codeword of weight w, the number of weight-j error vectors lying within radius t of that codeword is

(56) Ntnwj=∑a=aminamaxwan−wj−w+a,

where

$$a_{min}=max\left(0,w-j\right),$$

and

$$a_{max}=\min\left(w,\left\lfloor \frac{t+w-j}{2}\right\rfloor \right).$$

The RM-specific miscorrection bound is then

(57) $$B_{RM}\left(p\right)=\sum_{w=d_{min}}^{n}A_{w}\sum_{j=\max\left(0,w-t\right)}^{\min\left(n,w+t\right)}N_{t}(n,w,j)p^{j}{\left(1-p\right)}^{n-j}.$$

Thus,

(58) $$P_{miscorr}\leq B_{RM}\left(p\right),$$

and

(59) $$P_{UE}\leq B_{RM}\left(p\right)\varepsilon_{AMD}2^{-l}.$$

The relationship between the bounds is

(60) $$P_{miscorr}\leq B_{RM}\left(p\right)\leq B_{tail}\left(p\right),$$

where

(61) Btailp=∑j=t+1nnjpj1−pn−j.

**Theorem 3.**  *Miscorrection-based hybrid undetected error bound. Let* C=RM(r,m) *be used over* $BSC\left(p\right)$ *with a bounded-distance decoder of radius* t*. Let *$D_{mis}$ *denote the event that the decoder outputs an incorrect tagged candidate. Suppose the verification assumptions of Lemma 1 hold. Then, *

(62) $$P_{UE}\leq P_{miscorr}\varepsilon_{AMD}2^{-l}.$$

*If* $B_{RM}\left(p\right)$ *is the Reed–Muller decoding region bound defined in Equation (57), then* 

(63) $$P_{UE}\leq B_{RM}\left(p\right)\varepsilon_{AMD}2^{-l}.$$

*Moreover, *

(64) $$B_{RM}\left(p\right)\leq B_{tail}\left(p\right),$$

*so the code-specific result is no weaker than the generic correction-radius tail bound.*

**Proof.**  By definition,

$$P_{UE}=Pr\left(D_{mis}\right)Pr\left(A_{AMD}\cap A_{h}|D_{mis}\right).$$

Lemma 1 gives

$$Pr\left(A_{AMD}\cap A_{h}|D_{mis}\right)\leq \varepsilon_{AMD}2^{-l}.$$

Therefore,

$$P_{UE}\leq P_{miscorr}\varepsilon_{AMD}2^{-l}.$$

For a bounded-distance decoder, an incorrect output can occur only when the received vector enters the radius-t decoding region of a nontransmitted codeword. Summation over nonzero codewords, grouped according to the Reed–Muller weight distribution, yields the bound $B_{RM}\left(p\right)$. This proves Equation (63).

Finally, every miscorrection requires more than t channel errors, whereas not every error pattern of weight greater than t causes a miscorrection. Hence,

$$\left\{D_{mis}\right\}\subseteq \left\{W>t\right\},$$

which yields Equation (64). □

Empirical miscorrection refinement. If the exact RM weight enumerator or decoding region calculation is not available for every code, then it provides an empirical finite-length refinement.

Define

(65) $${\hat{P}}_{miscorr}=\frac{N_{mis}}{N_{MC}},$$

and

(66) $${\hat{P}}_{ver,acc|miscorr}=\frac{N_{UE}}{N_{mis}}.$$

Then,

(67) $${\hat{P}}_{UE}={\hat{P}}_{miscorr}{\hat{P}}_{ver,acc|miscorr}.$$

A semi-empirical upper estimate is

(68) $$B_{semi}={\hat{P}}_{miscorr}^{\;U}\varepsilon_{AMD}2^{-l},$$

where ${\hat{P}}_{miscorr}^{\;U}$ is the upper endpoint of a confidence interval for the empirical miscorrection probability.

This approach is useful because it separates:

- Looseness due to the decoder term;
- Looseness due to the verification term.

### 5.3. Reed–Muller Parameterization

Substituting the Reed–Muller parameters into Theorem 1 gives the explicit form

(69) Pue(r,m,p,l)≤∑i=2m−r−12m2mipi(1−p)2m−iεAMD 2−l,

where the lower summation index is equivalent to t+1 because $t=2^{m-r-1}-1$. This expression displays the dependence of the undetected error probability on all principal system parameters. The code order r, the code length exponent m, the BSC crossover probability p, the hash length l, and the AMD security parameter $\varepsilon_{AMD}$.

The interpretation is immediate. Increasing m increases block length and changes the binomial tail governing bounded-distance decoding failure. Decreasing r increases the Reed–Muller minimum distance and therefore enlarges the guaranteed correction radius. Increasing l decreases the seeded hash collision term exponentially. Strengthening the AMD construction decreases $\varepsilon_{AMD}$. Accordingly, outer-code design and verification-layer design remain mathematically separable while contributing jointly to the final integrity guarantee.

### 5.4. Comparison with Outer-Code-Only Protection

The same framework yields an immediate comparison with the corresponding outer-code-only architecture. If no AMD or hash verification is used, then the system-level undetected error probability is bounded only by the residual decoding-failure probability,

$$P_{ue}^{outer}\leq Pr\{w_{H}(E)>t\}.$$

Therefore, by Theorem 1,

$$P_{ue}^{hybrid}\leq P_{ue}^{outer}\;\varepsilon_{AMD}\;2^{-l}.$$

This shows that, within the specified model, the hybrid construction improves the outer-code-only bound by an explicit multiplicative factor

$$\varepsilon_{AMD}\;2^{-l}.$$

Whenever $\varepsilon_{AMD}<1\;a$ and l≥1, the hybrid scheme provides a strictly stronger upper bound than the corresponding outer-code-only architecture. This formalizes the main design advantage of the proposed layered construction.

### 5.5. Exponential Decay Regime

The explicit form of Theorem 1 also permits an asymptotic interpretation when the Reed–Muller order r is fixed and m→∞. In this regime,

$$n=2^{m},\;\;\frac{t}{n}=\frac{2^{m-r-1}-1}{2^{m}}=2^{-r-1}-2^{-m}.$$

Hence, for sufficiently large m, the correction threshold is asymptotically close to $2^{-r-1}n$. If the BSC crossover probability satisfies $p<2^{-r-1},$ then standard large-deviation estimates for the binomial tail imply exponential decay of $\;Pr\{w_{H}(E)>t\}$ in the block length n. Therefore, for fixed $\varepsilon_{AMD}$ and fixed l, the undetected error probability also decays exponentially in n. This is stated below.

**Corollary 1.**  *Fix* r*,* $\varepsilon_{AMD}$*, and* l*. If* $p<2^{-r-1},$ *then there exists a positive constant* c(r,p) *such that*

$$P_{ue}\leq e^{-c(r,p)n}\;\varepsilon_{AMD}\;2^{-l}$$

*for all sufficiently large* m*, where* $n=2^{m}$.

**Proof.**  By Theorem 1,

$$P_{ue}\leq Pr\{w_{H}(E)>t\}\varepsilon_{AMD}2^{-l}.$$

Since $w_{H}(E)∼Binomial(n,p)$ and $t/n\to 2^{-r-1}$, the assumption $p<2^{-r-1}$ implies that the tail probability $Pr\{w_{H}(E)>t\}$ decays exponentially in n by standard large-deviation bounds for binomial distributions. Multiplying by the fixed factor $\varepsilon_{AMD}2^{-l}$ yields the claim.

Corollary 1 shows that hybrid architecture preserves the exponential reliability gain associated with the outer code while adding verification-level suppression through the AMD and hash factors. In this sense, the outer code controls the large-deviation decay regime, whereas the inner verification layers shift the final undetected error probability downward by additional multiplicative factors. □

## 6. Simulation Validation

This section evaluates the proposed hybrid integrity protection framework under the binary symmetric channel BSC(p). The empirical implementation uses the original RM(r,m) configuration together with the GF(4)-based AMD construction and a 2-bit universal hash. To examine the influence of code parameters and verification strength more broadly, analytical parameter sweeps are additionally reported for several Reed–Muller codes, hash lengths, and AMD security levels.

The empirical and analytical results are kept separate. Empirical probabilities are reported only for the implemented RM(2, 5) system, whereas the additional Reed–Muller configurations are compared using the generic BSC correction-radius bound.

### 6.1. Simulation Configuration

For a binary Reed–Muller code RM(r,m) the block length, dimension, minimum distance, and bounded-distance decoding radius. Table 4 summarizes the Reed–Muller configurations considered in the analytical comparison.

The empirical baseline uses RM(2, 5) a 2-bit source message, the GF(4)-based AMD construction, and a seeded 2-bit universal hash. The remaining information positions are filled with zeros. Four protection modes are evaluated:

(70) $$T^{\left(O\right)}=M,$$

(71) $$T^{\left(A\right)}=\left(M,R,T_{AMD}\right),$$

(72) $$T^{\left(H\right)}=\left(M,S,T_{h}\right),$$

(73) $$T^{\left(AH\right)}=\left(M,R,T_{AMD},S,T_{h}\right).$$

These correspond to outer-code-only, AMD-assisted, hash-assisted, and hybrid protection, respectively. The principal validation parameters are listed in Table 5.

For each Monte Carlo trial, a source message is encoded, transmitted through BSC(p), and decoded. A decoder output is classified as correct, explicitly rejected, miscorrected and rejected by verification, or miscorrected and accepted. The last event is the system-level undetected error. The empirical undetected error probability is

(74) $${\hat{P}}_{UE}=\frac{N_{UE}}{N_{MC}},$$

where $N_{UE}$ is the number of undetected error events and $N_{MC}$ is the total number of trials. When no undetected event is observed, the result is interpreted as a finite-sample zero rather than as exact impossibility.

### 6.2. Empirical Results for RM(2,5)

**Figure 2:**
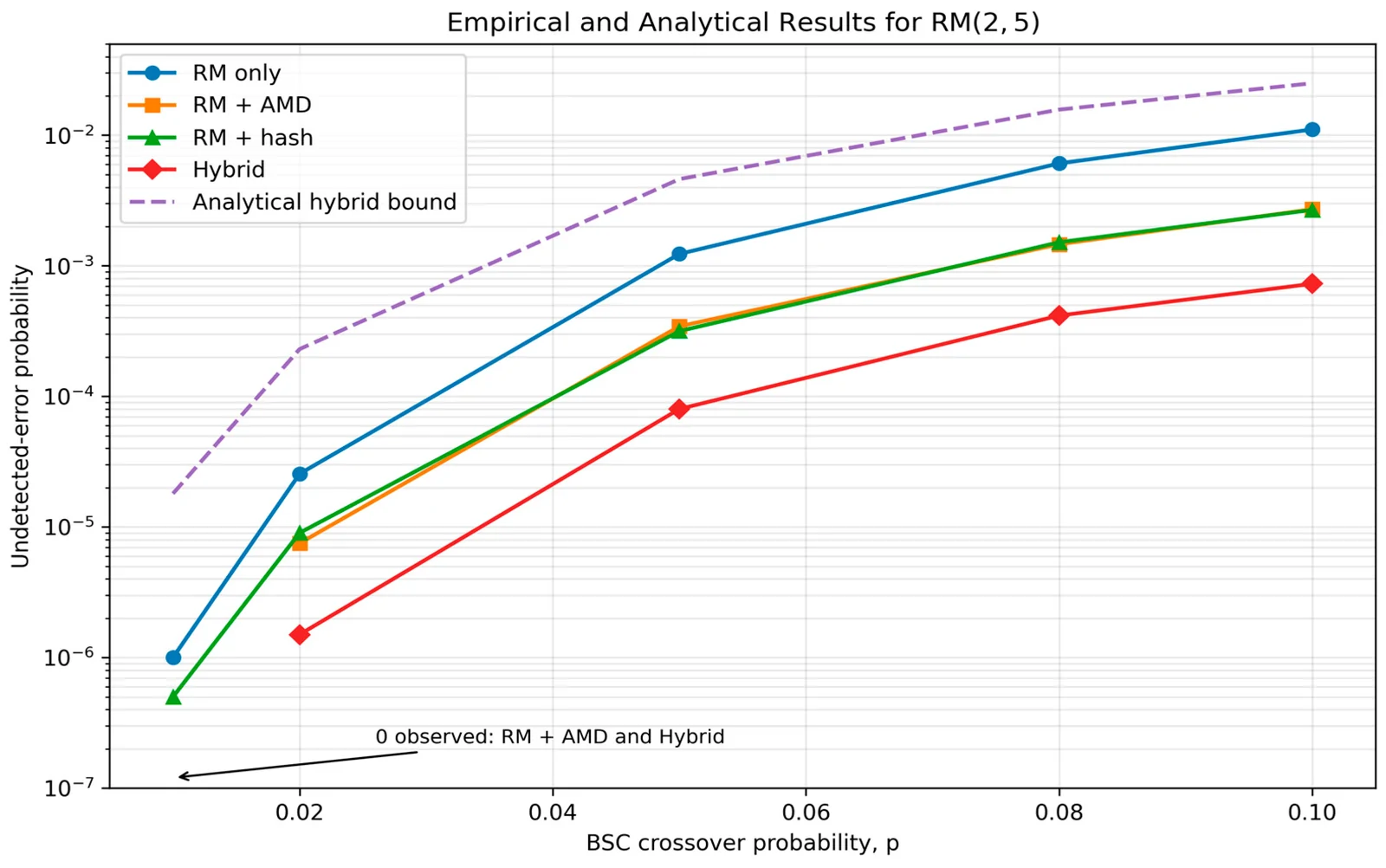
Empirical undetected error probability of the four protection modes for $RM\left(2,5\right)$, together with the analytical hybrid upper bound. Zero-event results are shown as finite-sample upper limits rather than exact zero probabilities.

Table 6 and Figure 2 report the empirical results for the implemented RM(2, 5) system. The decoder-error estimate varies slightly among protection modes because each mode uses a different subset of valid payloads inside the same outer code.

The outer-code-only mode produces the largest undetected error probability for every tested value of p, because any incorrect decoded source message is accepted automatically. Both single-layer verification modes reduce the probability substantially. The hybrid mode produces the smallest empirical undetected error probability at all nonzero observed points.

At p=0.05, for example, the hybrid probability is $8.0\times 10^{-5},$ compared with $1.232\times 10^{-3}\;$ for the outer-code-only mode. This corresponds to an improvement factor of approximately

$$\frac{1.232\times 10^{-3}}{8.0\times 10^{-5}}\approx 15.4.$$

At p=0.10, the corresponding improvement factor is

$$\frac{1.1068\times 10^{-2}}{7.28\times 10^{-4}}\approx 15.2.$$

The analytical bound remains above the measured hybrid probability for all tested channel conditions. The gap is expected because the bound uses the complete binomial tail beyond the guaranteed correction radius and worst-case verification-acceptance factors. It therefore includes channel patterns that may lead to explicit decoder rejection rather than miscorrection.

The zero entries at p=0.01 indicate that no undetected event was observed in the performed trials. They should not be interpreted as exact zero probabilities.

### 6.3. Influence of Verification Strength

The joint verification factor under the assumptions of Section 5 is $\varepsilon_{ver}=\varepsilon_{AMD}2^{-l}.$ Table 7 shows how this factor changes with the hash output length when $\varepsilon_{AMD}=2^{-4}.$

Each additional hash bit reduces the hash collision bound by a factor of two. Increasing l from two to 16 reduces the joint verification bound from approximately $1.56\times 10^{-2}$ to $9.54\times 10^{-7}$, at the cost of 14 additional hash bits. Table 8 shows the corresponding effect of the AMD security level for a fixed 4-bit hash.

Table 7 and Table 8 are analytical sensitivity results. They show the expected reduction in verification-acceptance probability but do not represent additional empirical AMD implementations.

### 6.4. Influence of Reed–Muller Parameters

For a bounded-distance decoder, an incorrect output can occur only when the channel-error weight exceeds t. The generic BSC-tail bound is

(75) Btailp=∑j=t+1nnjpj1−pn−j.

Using $\varepsilon_{AMD}=2^{-4},\;l=4,$ the generic hybrid bound becomes

(76) $$B_{hyb}\left(p\right)=2^{-8}B_{tail}\left(p\right).$$

Table 9 compares this bound across the Reed–Muller configurations in Table 4.

**Figure 3:**
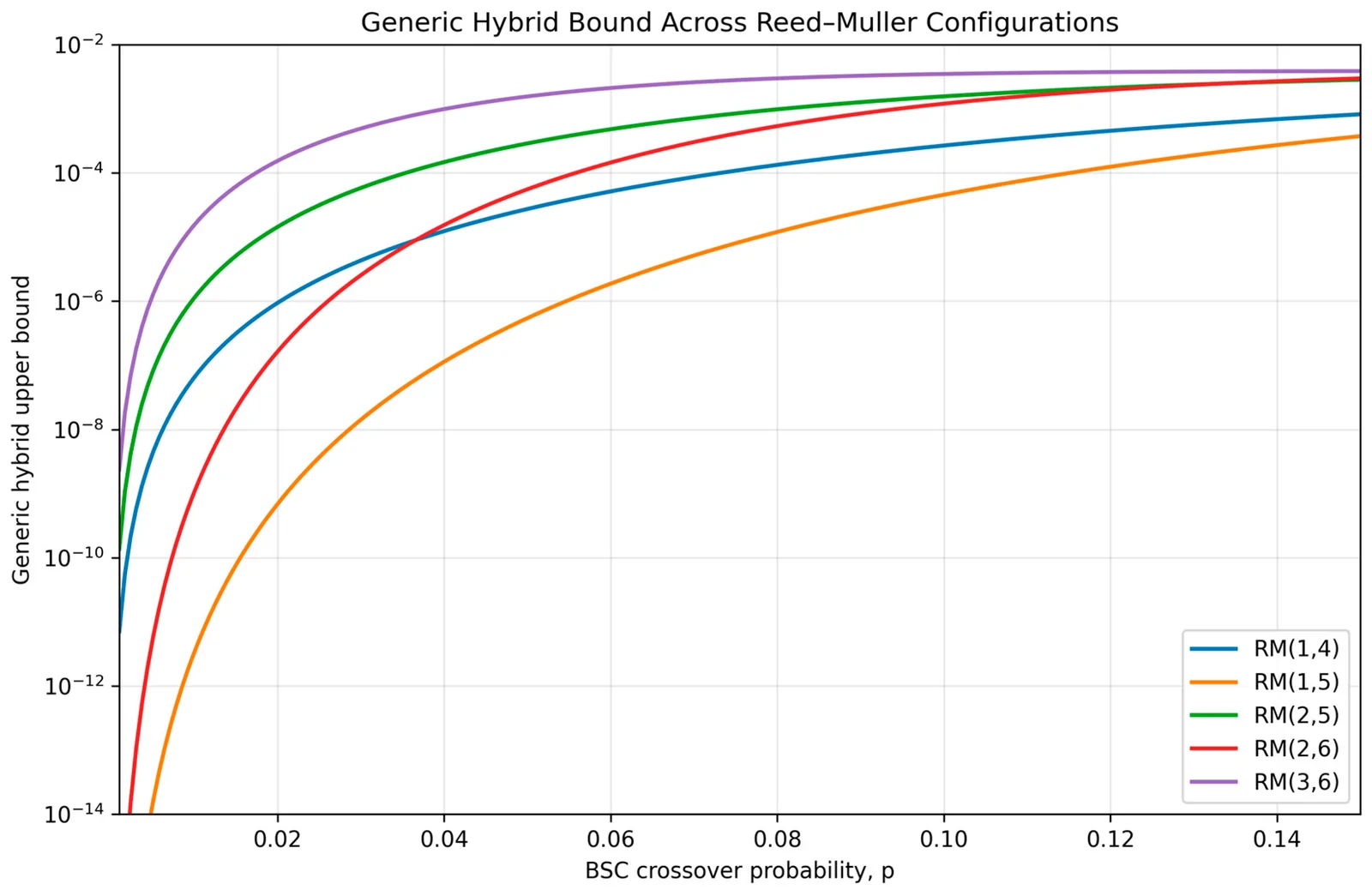
Generic hybrid upper bound versus BSC crossover probability for the evaluated Reed–Muller configurations. The curves illustrate the trade-off among code rate, minimum distance, and guaranteed correction radius.

The analytical comparison illustrates the expected rate–distance trade-off. The configurations RM(1, 5) and RM(2, 6), both with correction radius t=7, provide substantially smaller BSC tails than the configurations with t=3 at low and moderate crossover probabilities. However, this advantage is accompanied by lower code rates. For example, RM(1, 5) has the smallest analytical tail among the tested configurations but also the lowest rate, 6/32=0.1875. In contrast, RM(3, 6) has the highest rate, 42/64=0.6563, but the largest analytical tail at p=0.05 and p=0.10 (see Figure 3).

The values in Table 10 are upper bounds rather than measured decoder-miscorrection probabilities. They count all error patterns beyond the guaranteed correction radius, including patterns that may cause explicit decoding failure.

The $B_{tail}$ values and ratios should be recalculated from the final code rather than copied manually. The purpose of the table is to show that the largest source of looseness is usually the replacement of actual decoder miscorrection with the full correction-radius tail. For the current baseline, the verification factor is

$$\varepsilon_{AMD}2^{-l}=0.25\times 0.25=0.0625.$$

At p=0.05,

$$B_{tail}\approx 7.38\times 10^{-2},$$

while the empirical decoder-error estimate is approximately

$$2.99\times 10^{-3}.$$

Thus, the generic decoder term is approximately

$$\frac{7.38\times 10^{-2}}{2.99\times 10^{-3}}\approx 24.7$$

times larger than the measured decoder-error estimate. This already explains a substantial part of the final gap.

### 6.5. Redundancy Interpretation

The four protection modes differ in verification overhead. Let q denote the source-message length, $\rho_{AMD}$ the combined AMD randomness and tag length, s the hash-seed length, and l the hash output length. The total redundancy is

(77) $$\rho_{total}=\rho_{ver}+\rho_{pad}+\left(n-k\right),$$

where $\rho_{pad}$ denotes unused information positions.

The hybrid mode provides the strongest empirical protection in Table 11, but it also introduces the largest verification overhead. Consequently, its advantage should be interpreted as an integrity–redundancy trade-off rather than as an unconditional improvement.

The empirical RM(2, 5) results confirm the principal ordering predicted by the proposed model. Outer-code-only protection produces the highest undetected error probability, both single verification layers provide substantial reductions, and the combined hybrid mode produces the smallest measured probability.

The analytical parameter sweeps show that stronger AMD and hash parameters reduce the verification-acceptance bound multiplicatively under the assumptions of Section 5. They also show that Reed–Muller performance depends jointly on rate and correction radius. Low-rate configurations with larger t provide smaller BSC-tail bounds, whereas higher-rate configurations provide greater payload efficiency at the cost of weaker correction guarantees. The empirical evidence remains limited to the implemented $\mathrm{RM}\left(2,\;5\right)$ configuration. The additional Reed–Muller results in Table 9 are analytical comparisons and should not be interpreted as measured scalability results.

### 6.6. Complexity, Redundancy, and Fair-Budget Comparisons

Comparing only undetected error probability favors schemes with greater verification redundancy. The hybrid mode includes an AMD-protected representation, a hash seed, and a hash tag in addition to the redundancy introduced by the outer Reed–Muller code. Therefore, the four protection modes are also evaluated in terms of transmitted bits, effective source rate, computation, storage, and verification latency.

Let q denote the original source-message length, $\rho_{R}$ denote the AMD randomness length, $\rho_{T}$ denote the AMD tag length, s denote the universal hash seed length, l denote the hash output length, k denote the outer-code information dimension, n denote the transmitted codeword length, and $\rho_{pad}$ denote the number of unused information positions.

The AMD redundancy is

(78) $$\rho_{AMD}=\rho_{R}+\rho_{T},$$

and the hash redundancy is

(79) $$\rho_{h}=s+l.$$

The total verification redundancy is

(80) $$\rho_{ver}=\rho_{AMD}+\rho_{h},$$

while the total transmitted overhead relative to the original source message is

(81) $$\rho_{total}=\left(n-q\right).$$

Equivalently,

(82) $$\rho_{total}=\left(n-k\right)+\rho_{pad}+\rho_{ver}.$$

The effective source rate is

(83) $$R_{eff}=\frac{q}{n}.$$

These definitions ensure that fixed padding positions are counted as overhead rather than being omitted.

The exact bit values depend on the current GF(4)-based AMD and hash implementations. For the baseline system described in the manuscript, a 2-bit source message is used and the GF(4)-based AMD construction operates with one GF(4) randomness symbol and one GF(4) tag symbol. Since one GF(4) symbol corresponds to 2 bits, the baseline AMD representation contributes

$$\rho_{R}=2,\;\rho_{T}=2,\;\rho_{AMD}=4.$$

The manuscript also states that the hash has a 2-bit output. In the implemented hash family, the seed length is s=2 bits.

Under the baseline assumption $q=2,\;\rho_{AMD}=4,\;s=2,\;l=2,$ and using RM(2, 5) with k=16 and n=32, the payload and overhead are as follows (see Table 12).

This table reveals an important limitation of the original fixed-RM(2, 5) experiment: all modes transmit the same 32-bit codeword and protect the same 2-bit source message. Therefore, their total transmitted overhead is identical, but the outer-only mode wastes more information positions as fixed zero padding. The hybrid mode uses more of the available k=16 information positions for verification rather than increasing the transmitted block length. This is a fair comparison under the equal block length, equal source payload, and equal outer code (see Table 13). However, it is not a fair comparison under equal verification redundancy, because the outer-only mode has no post-decoding verification data.

The asymptotic order of the outer coding stage is unchanged by the verification layers. AMD and universal hashing introduce additive rather than multiplicative computational overhead. However, they consume information positions and require additional storage for randomness, seed, and tags.

Comparisons under equal block length and equal source payload show the effect of using available information positions for verification rather than padding (see Table 14). In the baseline $\mathrm{RM}\left(2,\;5\right)$ experiment, all modes transmit a 32-bit block and protect a 2-bit source message. Thus, the hybrid mode does not increase channel block length relative to the outer-only mode, but it uses more of the 16 information positions for integrity data. Comparisons with CRC-assisted schemes require a distinction between random error detection and adversarial manipulation detection. CRC is computationally efficient and provides strong practical detection of random error patterns, but it does not offer the same algebraic manipulation security definition as AMD. Universal hashing provides a probabilistic collision guarantee under its seed model, while the AMD layer provides a guarantee for the specified algebraic tampering family. Therefore, no single baseline dominates under every threat model.

BCH and Polar baselines are also decoder-dependent. Their performance must be reported together with decoder type, rate, block length, and verification overhead. Raw undetected error probability alone is insufficient for declaring one architecture superior.

## 7. Discussion

The theoretical results in Section 5 and the numerical results in Section 6 provide a consistent interpretation of layered integrity protection. In the proposed framework, the outer Reed–Muller code and the two verification layers address different parts of the failure process. The outer code acts at the transmission-decoding stage and reduces the probability that channel perturbations produce an incorrect decoded candidate. By contrast, the AMD and universal hash layers act after decoding and reduce the probability that such an incorrect candidate is accepted as valid. This functional separation is the key structural feature of the hybrid architecture and explains why the resulting undetected error probability is lower than that of the corresponding outer-code-only system.

A central outcome of the analysis is that the undetected error probability can be factorized into two conceptually distinct terms: a decoder-miscorrection term determined by the outer code and the channel, and a verification-acceptance term determined by the AMD and hash layers. In the fully specified model adopted in this paper, the first term is expressed through the binomial tail

$$Pr\{W_{H}(E)>t\},$$

where t is the bounded-distance decoding radius of the outer Reed–Muller code, while the second term is controlled by the product

$$\varepsilon_{AMD}2^{-l}.$$

This decomposition is mathematically useful because it isolates the contributions of coding and verification, making it possible to study their influence separately. In particular, the analysis shows that improving the outer code and strengthening the verification layers are not interchangeable operations: they affect different logical stages of the integrity protection process.

The simulation study supports this interpretation. For the instantiated finite-length system based on RM(2,5), the outer-only mode consistently produced the largest empirical undetected error probability, while the AMD-only and hash-only modes each yielded a substantial reduction. The combined hybrid mode achieved the smallest undetected error probability across all tested values of the channel crossover probability. This confirms the practical relevance of the factorized theoretical structure derived in Section 4. Although the analytical bound is conservative, the ordering of the empirical results agrees with the theorem and demonstrates that the layered screening principle remains effective in a concrete short-block implementation.

Another important point concerns the role of redundancy. In the present framework, total redundancy is naturally divided into verification redundancy and outer-code redundancy. This distinction is not merely notational. Outer-code redundancy increases the distance-based correction capability and therefore improves the probability that the correct tagged object reaches the verification stage. Verification redundancy, on the other hand, does not improve decoding itself, but reduces the probability that an incorrect decoded object is accepted. From a design perspective, this means that reliability improvement can be distributed across two independent mechanisms. In practical terms, once the outer code has reached a satisfactory correction capability, further reduction in undetected error probability may be achieved more efficiently by increasing the strength of the AMD or hash layer than by increasing the outer-code redundancy alone.

The paper also highlights the importance of using a fully specified model when analyzing hybrid integrity schemes. In the earlier version of the manuscript, the framework remained too abstract, and the numerical section was only illustrative. By fixing the channel to BSC(p), the outer code to RM(r,m), the decoder to bounded-distance decoding, and the verification mechanisms to explicit AMD and universal hash constructions, the analysis becomes both mathematically sharper and experimentally verifiable. This strengthens the contribution of the paper and makes the derived results reproducible.

The gap between the analytical upper bound and the empirical undetected error probability arises from several successive relaxations. First, the general theorem replaces the decoder-miscorrection event with the broader event that the number of channel errors exceeds the guaranteed correction radius. This binomial tail includes both miscorrections and explicit decoder failures. Second, the generic bound treats every error pattern beyond the correction radius as potentially leading to an incorrect accepted codeword, although many received vectors lie outside the bounded-distance region of every competing codeword. Third, the theorem applies the worst-case AMD acceptance probability to every incorrect candidate, even though the actual acceptance probability may be much smaller for the candidate distribution produced by the decoder. Fourth, the universal hash factor uses the worst-case collision bound $2^{-l}$, which need not be attained by the observed candidate pairs. Fifth, the AMD and hash bounds may each be maximized by different incorrect candidates, so their product can remain conservative even when the sequential conditional assumptions are valid. Finally, the generic theorem does not use the Reed–Muller weight distribution, coset structure, or exact decoding regions.

To address the first and final sources of looseness, the revised manuscript introduces a miscorrection-based representation and an RM-specific decoding region bound. The revised result replaces the BSC correction-radius tail with $P_{miscorr}$, or with the code-specific upper bound $B_{RM}\left(p\right)$. The remaining gap reflects the use of worst-case verification guarantees rather than the empirical distribution of incorrect decoded candidates.

### Case Reed–Muller Codes May Not Be the Optimal Choice

Although Reed–Muller codes provide an algebraically structured setting for the proposed finite-length integrity analysis, they are not universally optimal outer codes. Their available dimensions and rates are discrete, and some configurations may result in low effective payload rates or a substantial number of unused information positions, particularly for very short messages. For high-rate or high-throughput communication, LDPC, Polar, BCH, or other rate-flexible codes may provide a more favorable balance among error-correction performance, decoder complexity, and implementation efficiency. Reed–Muller codes may also be less attractive at long block lengths when near-maximum-likelihood, list, recursive-projection, or ensemble decoding is required, because such decoding methods can introduce considerable computational and latency costs. In soft-decision AWGN or fading channels, modern LDPC and Polar decoders may make more effective use of channel-reliability information than the hard-decision bounded-distance model considered in this study. Similarly, channels dominated by burst errors, insertion/deletion errors, or synchronization failures may be better served by interleaved BCH or Reed–Solomon codes, marker codes, or other channel-specific constructions. Reed–Muller coding may also be unnecessary in systems that already employ standardized CRC-assisted LDPC or Polar architectures. In such cases, introducing an RM decoder could increase implementation complexity without providing a proportional system-level benefit. The proposed AMD and universal hash verification layers are therefore not inherently restricted to Reed–Muller codes and may be combined with alternative outer codes. The present study uses Reed–Muller codes because their explicit parameters, algebraic structure, and finite-length decoding regions facilitate the analytical treatment, rather than because they are claimed to be optimal for every communication scenario.

At the same time, several limitations should be acknowledged. First, the theoretical bound in Theorem 1 is an upper bound and is not intended to be tight for every finite-length configuration. The simulation results indeed show that the measured hybrid failure probability lies well below the theorem bound, especially in low-noise regimes. Second, the finite-length validation in Section 5 is based on a short Reed–Muller code, namely RM(2,5), and therefore should be interpreted as a proof-of-concept validation rather than a large-scale performance study. Third, the specific AMD and hash instantiations used in the simulation were deliberately simple to make exhaustive finite-length validation feasible. More sophisticated AMD constructions or longer hash outputs would further reduce the empirical undetected error probability, but they would also increase verification redundancy. A further limitation is that the present work focuses on the binary symmetric channel and bounded-distance decoding. These assumptions were chosen to obtain an explicit and transparent analytical result, but they do not cover all practically relevant corruption models. In particular, the current analysis does not address erasures, burst errors, insertions, deletions, decoder mismatches, or adversarial channels with non-memoryless behavior. Likewise, only one outer-code family was considered. Reed–Muller codes are mathematically attractive because of their explicit parameters and classical structure, but the same layered verification principle could be studied with other code families as well. The multiplicative verification factor also depends on randomness and dependence assumptions. The product $\varepsilon_{AMD}2^{-l}$ is justified only when the AMD guarantee applies to the incorrect candidate and the fresh universal hash seed remains independent after conditioning on the decoder and AMD acceptance events. The bound may not hold under malicious seed selection, adaptive manipulation after observing the seed, compromised random-number generation, repeated seed reuse, or dependence between the AMD and hash verification data. Under such conditions, a conservative intersection bound based on the smaller marginal acceptance probability may be used if the marginal conditional guarantees remain valid. If even those guarantees fail, the analysis reduces to the decoder-error bound alone. Extending the model to adaptive seed-aware adversaries and reused verification randomness is left for future work.

Despite these limitations, the revised results establish a clear methodological contribution. The paper shows that hybrid integrity protection can be analyzed within a single mathematical framework in which the outer code governs residual decoding failure and the verification layers govern acceptance failure. This perspective clarifies why a layered architecture can outperform a standalone distance-based code even when both operate under the same outer-code parameters. It also provides a principled basis for parameter selection: the outer code should be chosen to control the residual decoding-failure region, whereas the AMD and hash layers should be chosen to control the acceptance probability of incorrect decoded candidates.

From a broader viewpoint, the proposed framework is relevant to systems in which accepted corruption is more critical than ordinary decoding failure. In such systems, the objective is not merely to reduce the number of erroneous outputs, but to reduce the probability that an erroneous output is treated as valid. The hybrid construction studied here is well suited to this objective because it introduces additional acceptance tests after decoding rather than relying entirely on distance-based protection.

Overall, the discussion confirms that the main value of the proposed approach lies in its layered mathematical structure. The outer Reed–Muller code, the AMD layer, and the universal hash layer perform complementary functions, and their joint use yields stronger protection against undetected error than any of the individual components alone. The analytical and numerical results together support the conclusion that hybrid coding with post-decoding verification is a viable and mathematically grounded strategy for high-assurance information integrity.

## 8. Conclusions

This paper developed a hybrid coding framework for information integrity based on the joint use of a binary Reed–Muller outer code, an algebraic manipulation detection layer, and a seeded universal hash verification layer. In contrast to purely distance-based protection, the proposed architecture separates two logically distinct tasks: the outer code reduces the probability that channel perturbations produce an incorrect decoded candidate, while the AMD and hash layers reduce the probability that such a candidate is accepted as valid. This separation makes the framework especially suitable for the analysis of undetected error events, which constitute the central reliability criterion in high-assurance integrity systems.

The present study does not claim novelty for the individual use of Reed–Muller coding, AMD, universal hashing, or bounded-distance decoding. Its contribution lies in connecting these established mechanisms through a formally specified finite-length acceptance model. In this model, the outer decoder determines whether an incorrect tagged candidate is produced, whereas the verification layers determine whether that candidate is accepted. The principal coding-theoretic refinement is the replacement of the generic correction-radius tail by a Reed–Muller-specific miscorrection expression based on the code’s weight distribution and bounded-distance decoding regions. This refinement separates channel patterns that merely exceed the guaranteed correction radius from those that actually reach the decoding sphere of an incorrect codeword. Consequently, it provides a more informative finite-length integrity bound and clarifies how Reed–Muller structure affects the final probability of undetected acceptance. The numerical results should be interpreted as finite-length evidence for the stated models and parameter settings rather than as proof that Reed–Muller codes are universally optimal for integrity protection. The appropriate outer code depends on rate, block length, channel, decoder, latency, and implementation constraints. Reed–Muller codes are useful here because their algebraic structure and finite-length spectra permit a transparent code-specific analysis; BCH, Polar, LDPC, and CRC-assisted alternatives may be preferable in other operating regimes. The analytical results provide conservative integrity guarantees rather than exact performance predictions. The generic bound is simple and parameterized by r, m, p, l, and $\varepsilon_{AMD}$, while the refined result incorporates decoder miscorrection and Reed–Muller decoding region information. The empirical results confirm the expected ordering of the protection modes, but the numerical gap shows that worst-case system-level bounds should not be interpreted as tight estimates of finite-length performance.

Another important conclusion is that the proposed framework admits a meaningful redundancy decomposition. Outer-code redundancy and verification redundancy play different roles and can therefore be tuned separately. The former determines the distance-based correction capability of the code, while the latter governs the acceptance probability of incorrect decoded candidates. This provides useful design flexibility and suggests that, once the outer code has reached a suitable correction regime, further improvements in undetected error performance may be achieved efficiently by strengthening the verification layers. The revised formulation of the paper also clarifies the mathematical novelty of the approach. Rather than presenting a generic architecture, the study analyzes a concrete hybrid model in which coding and post-decoding verification are integrated into a single probabilistic framework. This makes the contribution more rigorous, more reproducible, and more appropriate for a mathematics-oriented treatment of integrity protection.

Several directions remain open for future work. First, the present analysis could be extended to longer Reed–Muller codes and to additional code families with different distance and decoding properties. Second, stronger AMD constructions and larger universal hash outputs could be investigated to study the trade-off between verification redundancy and empirical performance. Third, the current channel model could be generalized to include burst errors, erasures, insertions, deletions, or adversarial perturbation models. Finally, it would be valuable to derive sharper finite-length bounds and asymptotic trade-off results for optimal allocation between outer-code redundancy and verification redundancy.

In summary, the results of this paper show that combining outer Reed–Muller coding with AMD-based and universal hash-based post-decoding verification provides a mathematically coherent and practically effective strategy for reducing undetected error probability. The proposed hybrid framework therefore offers a useful foundation for the design and analysis of high-assurance information-integrity systems.

## Figures and Tables

**Table 1:** Positioning of the proposed study relative to representative integrity protection approaches.

Approach or Literature Stream	Outer Protection	Verification Mechanism	Undetected Error Treatment	Finite-Length Evaluation	Redundancy or Overhead Analysis
Classical undetected error analysis of linear codes	General linear block code	Code membership or syndrome-based detection	Derived from code weight distribution or decoding regions	Yes	Usually coding redundancy only
Combined error correction and error detection	General linear code with reduced correction radius or unused syndromes	Remaining syndromes or additional detection rule	Code-specific finite-length expression		Correction–detection trade-off
CRC-assisted coded systems	BCH, convolutional, Polar, LDPC, or other outer code	CRC	Often empirical or code/decoder-specific	Commonly included	CRC and coding overhead
AMD-based integrity systems	Optional or application-dependent	AMD code	Bounded by AMD security parameter	Construction-dependent	AMD randomness and tag overhead
Universal hash-based verification or authentication	Optional or application-dependent	Universal or almost-universal hash	Collision-probability bound		Seed and tag overhead
Natural and malicious fault protection	Error-control or error-detection code	Code-based masking or integrity check	Error-masking or acceptance probability	Application-dependent	Often implementation-specific
Present work	Binary *RM*(*r,m*) with bounded-distance decoding	AMD plus seeded 2-universal hashing	Generic bound and refined RM-spectrum-based finite-length bound	Multiple RM and verification parameters	Coding, tag, seed, complexity, and matched-budget analysis

**Table 2:** Threat model and principal assumptions of the proposed hybrid integrity protection system.

Element	Assumption Adopted in This Study	Role in the System
Source message	A binary source message *M* is augmented with AMD and universal hash verification information before outer coding.	Defines the information object that must be recovered and authenticated.
Outer code	A public binary Reed–Muller code RM(r,m) is used.	Provides distance-based correction of stochastic bit errors.
Channel model	The primary analytical channel is a memoryless *BSC*(*p*), in which each transmitted bit is independently flipped with probability *p*.	Models random non-adversarial transmission or storage errors.
Decoder	The receiver uses a public bounded-distance decoder with correction radius *t* = [(*d_min_*/−1)/2].	Corrects all error patterns of weight at most *t*; beyond this radius, failure or miscorrection may occur.
Residual incorrect candidate	An incorrect candidate may arise when the channel-error pattern lies outside the guaranteed correction radius.	Represents the input to the post-decoding integrity checks.
AMD threat	The AMD model permits a nonzero admissible additive modification of the AMD-protected representation.	Detects algebraically structured manipulation except with probability bounded by the AMD security parameter.
Adversary knowledge	The adversary may know the Reed–Muller code, decoding algorithm, AMD construction, hash family, and overall system architecture.	Ensures that protection is not based on algorithm secrecy.
AMD randomness	AMD randomness is freshly generated according to the selected AMD construction and is independent of the source message unless the construction specifies otherwise.	Enables the stated AMD acceptance bound.
Universal hash family	A public seeded 2-universal hash family with (\ell)-bit output is used.	Provides an explicit collision-probability bound for distinct messages.
Hash seed	A fresh seed is selected independently of the source message and AMD randomness. It is transmitted as part of the tagged object and is not assumed to be secret.	Allows the receiver to reproduce the hash while preserving the universal hashing experiment.
Relationship between threats	Stochastic channel corruption occurs before decoding; AMD and hash checks are applied to the decoded candidate. An extended application may also include manipulation at an untrusted relay or storage stage.	Separates decoding reliability from post-decoding acceptance integrity.
Acceptance rule	A candidate is accepted only if decoding produces a syntactically valid tagged representation and all enabled AMD and hash checks succeed.	Defines the system-level integrity decision.
Rejection rule	The candidate is rejected if decoding fails, the tagged representation is invalid, or at least one verification check fails.	Converts detected corruption into a safe failure rather than silent acceptance.
Undetected error event	An incorrect source-message candidate is output and accepted as valid.	Defines the principal performance metric.
Scope limitation	The theorem does not model adaptive adversarial control of the BSC, compromised randomness, malicious seed selection, seed reuse, or arbitrary insertion/deletion attacks.	Delimits the validity of the theoretical results.

**Table 3:** Principal notation used throughout the manuscript.

Symbol	Definition
F_2_	Binary field
M	Original source message
$\hat{M}$	Candidate source message recovered by the receiver
R	Fresh randomness used by the AMD encoder
S	Seed selecting a hash function from the universal hash family
T_AMD_	AMD verification data or AMD tag associated with M
T_h_	Universal hash tag, $T_{H}=h_{S}\left(M\right)$
T	Complete tagged representation before outer encoding
$\hat{T}$	Candidate tagged representation produced by the decoder
C	Binary Reed–Muller code used as the outer code
RM(r, m)	Binary Reed–Muller code of order (r) and length exponent (m)
r	Order of the Reed–Muller code
m	Number of Boolean variables defining the Reed–Muller code
n	Reed–Muller block length, $n=2^{m}$
K	Reed–Muller dimension
d_min_	Minimum Hamming distance, $d=2^{m-r}.$
t	Guaranteed bounded-distance decoding radius
X	Transmitted Reed–Muller codeword
E	Binary channel-error vector
Y	Received vector
p	Crossover probability of the binary symmetric channel
l	Output length, in bits, of the universal hash
$E_{AMD}$	Maximum AMD acceptance probability under the stated manipulation model
D_fail_	Event that the decoder declares failure and outputs no candidate
D_mis_	Event that the decoder outputs an incorrect candidate
A_AMD_	Event that the candidate passes AMD verification
A_h_	Event that the candidate passes universal hash verification
R_rej_	Event that the candidate is rejected by at least one verification check
U	System-level undetected error event
P_UE_	Probability of undetected error
⊥	Explicit rejection or decoder-failure output

**Table 4:** Reed–Muller configurations considered in the validation.

Code	n	k	Rate k/n	d_min_	t
RM(1, 4)	16	5	0.3125	8	3
RM(1, 5)	32	6	0.1875	16	7
RM(2, 5)	32	16	0.5000	8	3
RM(2, 6)	64	22	0.3438	16	7
RM(3, 6)	64	42	0.6563	8	3

**Table 5:** Empirical and analytical validation settings.

Parameter	Setting
Main channel	Memoryless BSC(p)
Empirical crossover probabilities	0.01, 0.02, 0.05, 0.08, 0.10
Empirical RM code	RM(2, 5)
Analytical RM codes	All codes listed in Table 4
Source-message length	2 bits
Baseline AMD construction	GF(4)-based AMD
Baseline AMD bound	$\varepsilon_{AMD}=2^{-2}=0.25$
Baseline hash length	*l* = 2 bits
Baseline hash bound	$2^{-l}=0.25$
Baseline joint verification factor	0.0625
Decoder	Bounded-distance decoder
Protection modes	Outer-only, AMD-assisted, hash-assisted, hybrid

**Table 6:** Empirical undetected error probabilities for RM(2, 5).

*p*	Decoder-Error Estimate	Outer-Only	AMD-Assisted	Hash-Assisted	Hybrid	Analytical Hybrid Bound
0.01	1.5 × 10^−6^–4.5 × 10^−6^	1.0 × 10^−6^	0 observed	5.0 × 10^−7^	0 observed	1.80 × 10^−5^
0.02	7.15 × 10^−5^	2.55 × 10^−5^	7.5 × 10^−6^	9.0 × 10^−6^	1.5 × 10^−6^	2.30 × 10^−4^
0.05	2.99 × 10^−3^	1.232 × 10^−3^	3.44 × 10^−4^	3.16 × 10^−4^	8.0 × 10^−5^	4.61 × 10^−3^
0.08	1.391 × 10^−2^	6.084 × 10^−3^	1.462 × 10^−3^	1.514 × 10^−3^	4.16 × 10^−4^	1.57 × 10^−2^
0.10	2.476 × 10^−2^	1.1068 × 10^−2^	2.712 × 10^−3^	2.680 × 10^−3^	7.28 × 10^−4^	2.50 × 10^−2^

**Table 7:** Joint verification bound versus hash output length.

*l*	$2^{-l}.$	$\varepsilon_{AMD}$	$\varepsilon_{AMD}2^{-l}$
2	2.500000 × 10^−1^	6.250000 × 10^−2^	1.562500 × 10^−2^
4	6.250000 × 10^−2^		3.906250 × 10^−3^
8	3.906250 × 10^−3^		2.441406 × 10^−4^
16	1.525879 × 10^−5^		9.536743 × 10^−7^

**Table 8:** Joint verification bound versus AMD security level for l=4.

$\varepsilon_{AMD}$	$2^{-4}$	Joint Verification Bound
2^−2^ = 0.25	0.0625	1.562500 × 10^−2^
2^−4^ = 0.0625		3.906250 × 10^−3^
2^−8^ = 0.00390625		2.441406 × 10^−4^

**Table 9:** Generic BSC-tail and hybrid bounds for different Reed–Muller codes.

Code	*p*	$B_{tail}\left(p\right)$	$B_{tail}\left(p\right)$
RM(1, 4)	0.01	1.653064 × 10^−5^	6.457280 × 10^−8^
	0.05	7.003908 × 10^−3^	2.735901 × 10^−5^
	0.10	6.840617 × 10^−2^	2.672116 × 10^−4^
RM(1, 5)	0.01	8.491876 × 10^−10^	3.317139 × 10^−12^
	0.05	1.390820 × 10^−4^	5.432892 × 10^−7^
	0.10	1.168545 × 10^−2^	4.564631 × 10^−5^
RM(2, 5)	0.01	2.874736 × 10^−4^	1.122944 × 10^−6^
	0.05	7.380549 × 10^−2^	2.883027 × 10^−4^
	0.10	3.996941 × 10^−1^	1.561305 × 10^−3^
RM(2, 6)	0.01	2.688865 × 10^−7^	1.050338 × 10^−9^
	0.05	1.421864 × 10^−2^	5.554158 × 10^−5^
	0.10	3.078292 × 10^−1^	1.202458 × 10^−3^
RM(3, 6)	0.01	3.943524 × 10^−3^	1.540439 × 10^−5^
	0.05	3.985907 × 10^−1^	1.556995 × 10^−3^
	0.10	8.937088 × 10^−1^	3.491050 × 10^−3^

**Table 10:** Decomposition and tightness of the hybrid bound for RM(2, 5).

*p*	BSC Tail *B_tail_*	Empirical Decoder-Error Estimate	Empirical *P_UE_*	Generic Hybrid Bound	Ratio: Generic Bound
0.01	2.8747 × 10^−4^	1.5 × 10^−6^–4.5 × 10^−6^	0 observed	1.80 × 10^−5^	Not defined for zero estimate
0.02	3.68 × 10^−3^	7.15 × 10^−5^	1.5 × 10^−6^	2.30 × 10^−4^	153.3
0.05	7.3805 × 10^−2^	2.99 × 10^−3^	8.0 × 10^−5^	4.61 × 10^−3^	57.6
0.08	2.512 × 10^−1^	1.391 × 10^−2^	4.16 × 10^−4^	1.57 × 10^−2^	37.7
0.10	3.9969 × 10^−1^	2.476 × 10^−2^	7.28 × 10^−4^	2.50 × 10^−2^	34.3

**Table 11:** Redundancy structure of the protection modes.

Mode	Protected Payload Length	Verification Redundancy
Outer-code-only	*q*	0
AMD-assisted	*q* + *p_AMD_*	*p_AMD_*
Hash-assisted	*q* + *s* + *l*	*s* + *l*
Hybrid	*q* + *p_AMD_* + *s* + *l*	*p_AMD_* + *s* + *l*

**Table 12:** Redundancy and effective-rate comparison for the baseline RM(2,5) implementation.

Mode	Source Bits (q)	AMD Bits	Seed Bits	Hash Bits	Used Information Positions	Padding Positions	Outer Parity Bits (n − k)	Total Overhead (n − q)	Effective Rate (q/n)
RM only	2	0	0	0	2	14	16	30	0.0625
RM + AMD	2	4	0	0	6	10	16	30	0.0625
RM + hash	2	0	2	2	6	10	16	30	0.0625
RM + AMD + hash	2	4	2	2	10	6	16	30	0.0625

**Table 13:** Asymptotic computational and storage cost of the evaluated protection modes.

Mode	Outer Encoding	Outer Decoding	Additional Generation and Verification Cost	Additional Transmitted Data	Additional Receiver Storage
RM only	*C_RM,enc_*(*n*)	C_RM,dec_(n)	None	None beyond outer code	Code and decoder state
RM + AMD			*O*(*q* + *p_R_*)	*p_R_* + *p_T_* bits	AMD parameters and temporary field symbols
RM + hash			*O*(*q*)	*s* + *l* bits	Hash seed and tag
Hybrid			*O*(*q* + *p_R_*) + *O*(*q*)	*p_R_* + *p_T_* + *s* + *l* bits	AMD parameters, seed, and tag

**Table 14:** Structure of the fair-budget comparison.

Scheme	Outer Code	Decoder	Verification Bits	Block Length	Effective Rate	Integrity Model
RM only	RM(2, 5)	Bounded- distance	0	32	0.0625	No post-decoding check
RM + CRC-4			4			CRC error detection
RM + AMD			4			Algebraic manipulation detection
RM + hash-4			Seed + 4-bit tag			Universal hash collision bound
RM + AMD + hash			AMD + seed + tag			Combined verification
BCH + CRC-4	Shortened BCH, approximately 31–32 bits	Algebraic BCH decoder	4			CRC-assisted block coding
Polar + CRC-4	Polar, (N = 32)	SCL decoder	4			CRC-assisted list decoding

## Data Availability

Data are contained within the article.
